# The Japan Society of Ultrasonics in Medicine guidelines on non-mass abnormalities of the breast

**DOI:** 10.1007/s10396-023-01308-9

**Published:** 2023-06-01

**Authors:** Toshikazu Ito, Ei Ueno, Tokiko Endo, Kiyoka Omoto, Akira Kuwajima, Nobuyuki Taniguchi, Hiroko Tsunoda, Eriko Tohno, Hideyuki Hashimoto, Yasuhisa Fujimoto, Takanori Watanabe

**Affiliations:** 1 Division of Breast and Endocrine Surgery, Department of Surgery, Faculty of Medicine, Kindai University, Osaka, Japan; 2 Tsukuba International Breast Clinic, Ibaraki, Japan; 3 Department of Breast Surgery, National Hospital Organization Higashinagoya National Hospital, Aichi, Japan; 4 Department of Laboratory Medicine, Saitama Medical Center, Jichi Medical University, Saitama, Japan; 5 Department of Health Check-Up, PL Tokyo Health Control Center, Tokyo, Japan; 6 Department of Clinical Laboratory Medicine, Jichi Medical University, Tochigi, Japan; 7 Department of Radiology, St. Luke’s International Hospital, Tokyo, Japan; 8 Medical Department, Chiba Foundation for Health Promotion and Disease Prevention, Chiba, Japan; 9 Breast Center, Suita Tokushukai Hospital, Osaka, Japan; 10 Department of Breast Surgery, National Hospital Organization Sendai Medical Center, Miyagi, Japan

**Keywords:** Non-mass abnormalities, Breast ultrasound, Hypoechoic area, Abnormalities of the ducts, Architectural distortion

## Abstract

It is possible to appropriately diagnose non-mass abnormalities by elucidating ultrasound non-mass abnormality findings and sharing the concept. If non-mass abnormalities can be diagnosed early, the number of curable cases could increase, leading to fewer breast cancer deaths. The Japan Society of Ultrasonics in Medicine (JSUM) Terminology/Diagnostic Criteria Committee has classified non-mass abnormalities into five subtypes: hypoechoic area in the mammary gland, abnormalities of the ducts, architectural distortion, multiple small cysts, and echogenic foci without a hypoechoic area. We herein define the findings for each of these subtypes and present a summary of the JSUM guidelines on non-mass abnormalities of the breast generated based on those findings.

## Introduction

With respect to non-mass image-forming breast cancer on breast ultrasound (US), lesions that are difficult to recognize began to be identified as masses presenting a variety of morphologies, including the visualization of breast cancer with abnormal nipple discharge as mammary duct dilatation on US images in the 1980s [1, 2]. In addition, with the widespread adoption of mammography screening, many cases of breast cancer showing microcalcifications are being detected [3–6]; the morphology has been identified by collating lesions on mammography images with US images, and it is now possible to detect the US image characteristics of non-mass image-forming breast cancer [7, 8]. The subsequent widespread adoption of US breast screening as well as advances in diagnostic equipment have resulted in an increasing awareness of non-mass abnormalities [9–24].

The Breast Imaging Reporting and Data System (BI-RADS) 5th edition US lexicon includes masses, calcifications, and associated features, but it does not include the concept of non-mass abnormalities [25, 26].

Non-mass abnormalities present a variety of morphologies and findings on US images, but in the case of malignant lesions, not only ductal carcinoma in situ (DCIS) but also invasive lobular carcinoma (ILC), invasive ductal carcinoma (IDC), and other carcinomas present non-mass abnormality findings [21, 23, 26–36]. Appropriate diagnosis of invasive cancers presenting as non-mass abnormalities may help reduce breast cancer deaths; therefore, it is important to diagnose them by assessing US imaging findings and their distribution.

The Japan Society of Ultrasonics in Medicine (JSUM) Terminology/Diagnostic Criteria Committee has classified non-mass abnormalities into five subtypes: hypoechoic area in the mammary gland, abnormalities of the ducts, architectural distortion, multiple small cysts, and echogenic foci without a hypoechoic area. We herein define the findings for each of these subtypes and present a summary of the JSUM guidelines on non-mass abnormalities of the breast generated based on the findings that correspond to each subtype.

## Definition of non-mass abnormalities

Non-mass abnormalities refer to lesions that are difficult to discern as masses on US images. Note that for a certain period of time when the concept of the term was first proposed, "non-mass image-forming lesion" was used, but the terminology has since been reexamined and is now unified as "non-mass abnormalities".

Note that a breast "mass" refers to a space-occupying lesion believed to be a lump formed by components that differ from the surrounding tissue, and corresponds to "mass image-forming lesion" in Guidelines for Ultrasound Diagnosis of Breast Disorders (2005) [9–11].

## Classification of non-mass abnormalities

### Hypoechoic area in the mammary gland

Definition: A hypoechoic area with properties that differ from those of the surrounding mammary gland or contralateral mammary gland, and which is difficult to discern as a mass.

The term "hypoechoic" used for non-mass abnormalities refers to an echo level that can be recognized as being lower than that of the surrounding mammary gland.

Note that the term "hypoechoic" used to express the echo level in a breast mass is generally based on the echo level of fat; therefore, it differs from the standard used for non-mass abnormalities.

In actual cases, it is sometimes difficult to assess whether a finding is a hypoechoic area or a mass, but there is no particular problem with the examiner or diagnostician subjectively making either assessment during the diagnosis process. Either way, the diagnosis should fundamentally be made with intraductal proliferative lesions in mind, both benign and malignant.

The differential diagnosis will primarily include, for example, DCIS and IDC or ILC with extensive intraductal component (EIC) in the case of malignant lesions, and ductal hyperplasia, adenosis, and sclerosing lesions in the case of benign lesions.

A hypoechoic area in the mammary gland is further classified into the following three subtypes, but not all can be distinctly classified.

**Fig. 1:**
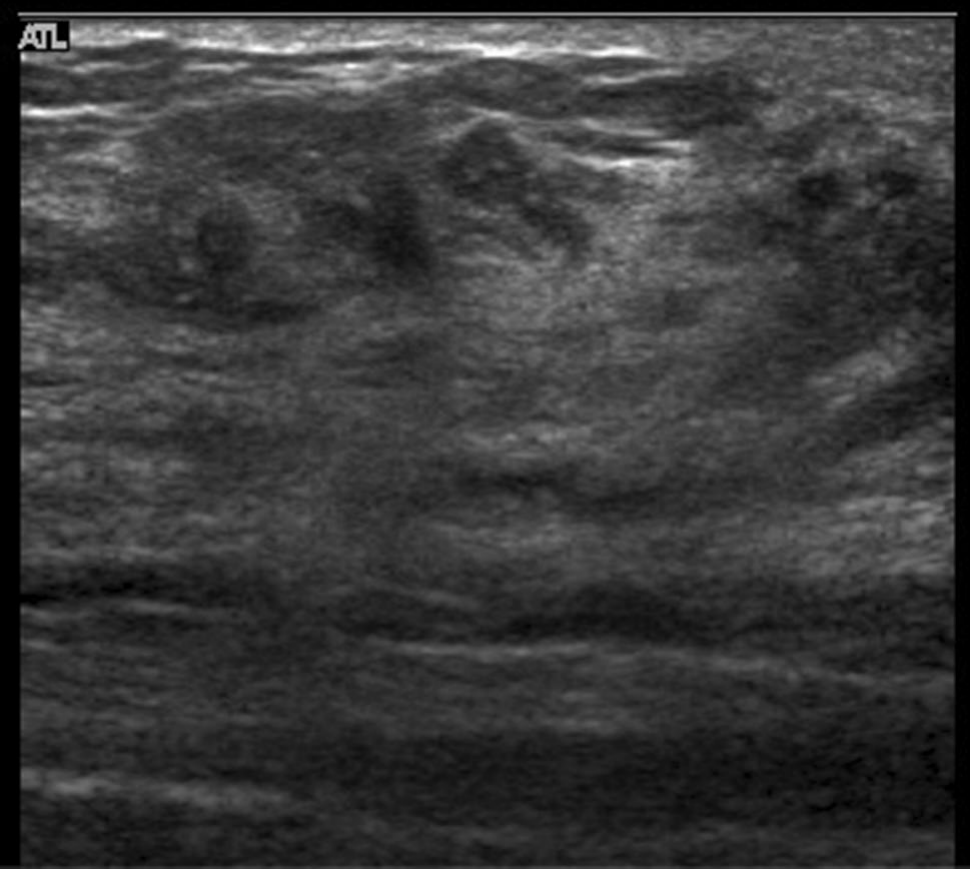
Case of ductal carcinoma in situ (DCIS). Ultrasound shows a patchy hypoechoic area in the mammary gland

**Fig. 2:**
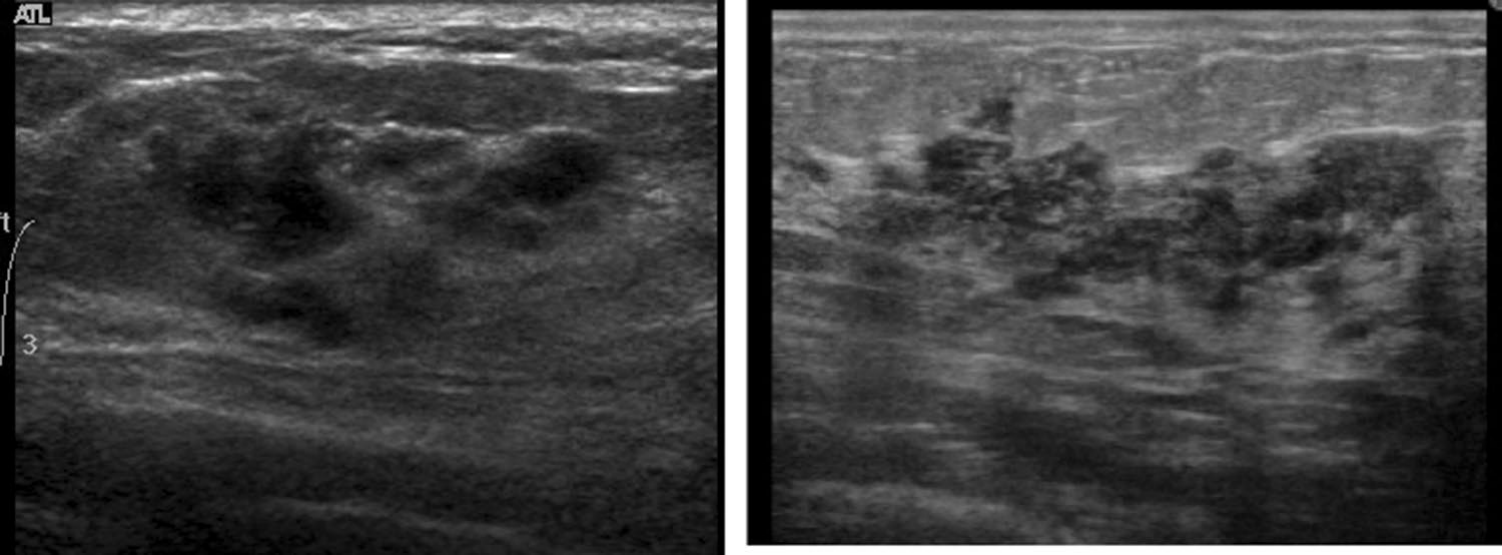
Two cases of ductal carcinoma in situ (DCIS). Ultrasound shows geographic hypoechoic areas in the mammary gland

**Fig. 3:**
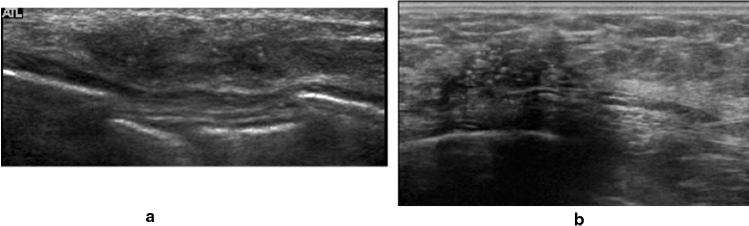
Two cases of ductal carcinoma in situ (DCIS). Ultrasound shows indistinct hypoechoic areas in the mammary gland

① **Patchy or mottled hypoechoic area:** There are multiple relatively small hypoechoic areas that as a whole can be recognized as one lesion (Fig. 1).
② **Geographic hypoechoic area:** It looks as if a patchy or mottled hypoechoic area has fused together (Fig. 2).
③ **Indistinct or ill-defined hypoechoic area:** It is difficult to express as either patchy/mottled or geographic, and it cannot be recognized as a mass due to having an indistinct or ill-defined border (Fig. 3a).

A segmental or focal hypoechoic area in the mammary gland indicates malignant potential. If echogenic foci suggestive of calcification are found in a hypoechoic area, the malignant potential is higher (Fig. 3b). If blood flow is clearly increased in a hypoechoic area with thickening of the mammary gland, the presence of intraductal proliferative changes should be kept in mind when making the diagnosis.

### Abnormalities of the ducts

Definition: The properties of the ducts such as caliber, wall thickness, or regularity are different from those of normal ducts.

Abnormalities of the ducts are classified into the following three types.

**Fig. 4:**
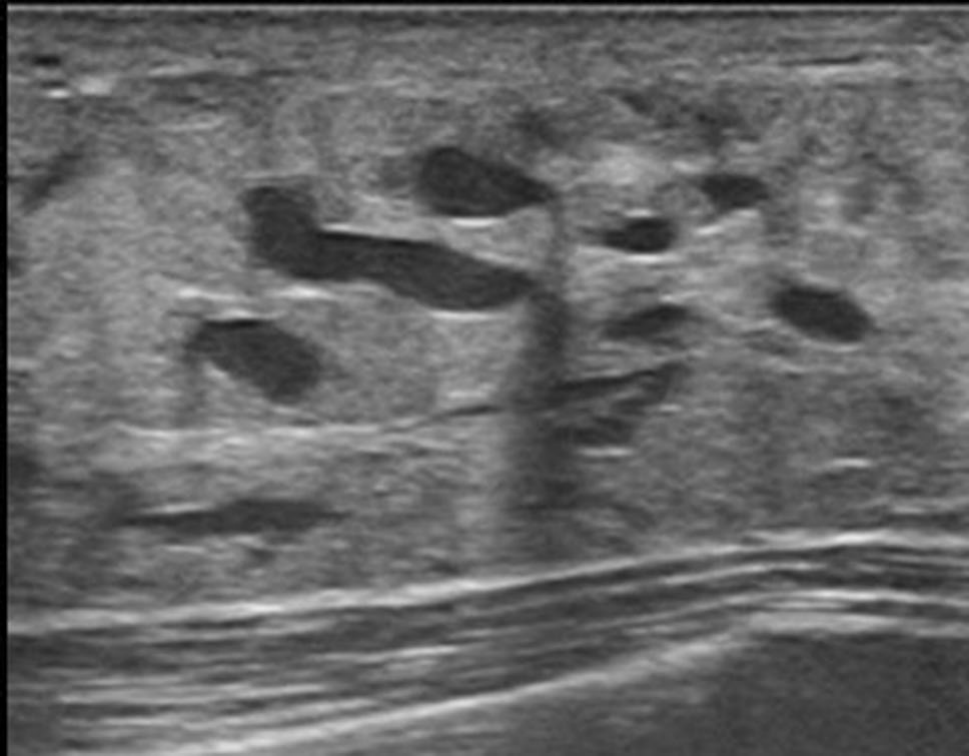
Ultrasound shows duct dilatation, but it is not considered an abnormality based on a finding of duct dilatation alone during late gestation or lactation

① **Duct dilatation:** A state in which ducts are clearly dilated beyond the extent of the areola as compared with other ducts.

(Explanation) Ducts within the extent of the areola are not considered an abnormality based on a finding of dilatation alone as dilatation is also found in normal cases. Moreover, it is not considered an abnormality based on a finding of duct dilatation alone during late gestation or lactation (Fig. 4). Note that the bilateral or diffuse linear high echoes sometimes seen in the mammary glands of young individuals are believed to be ducts and are not considered an abnormality.

② **Ducts with internal echoes:** The following are examples of US findings seen in the ducts.

- Solid echoes: A finding that suggests a solid lesion such as a hypoechoic component seen in the ducts.
- Echogenic foci: Hyperechoic spots in the ducts.
- Floating echoes: A finding in which breast milk, abscess, blood, or other liquid is seen in the ducts and its fluidity is observed.

**Fig. 5:**
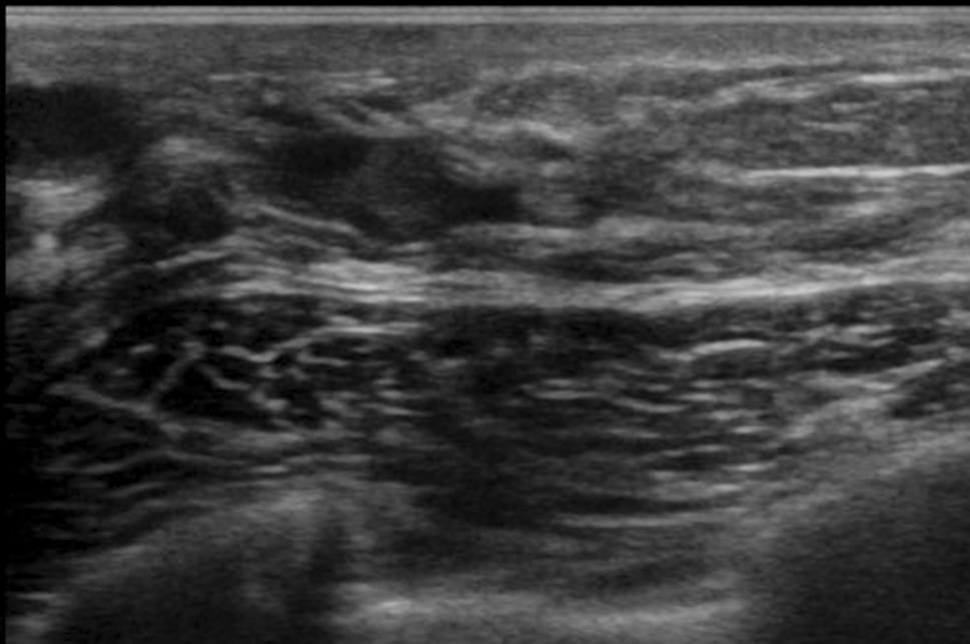
Case of intraductal papilloma. Ultrasound shows that the beginning of the solid component within dilated ducts is precipitous

**Fig. 6:**
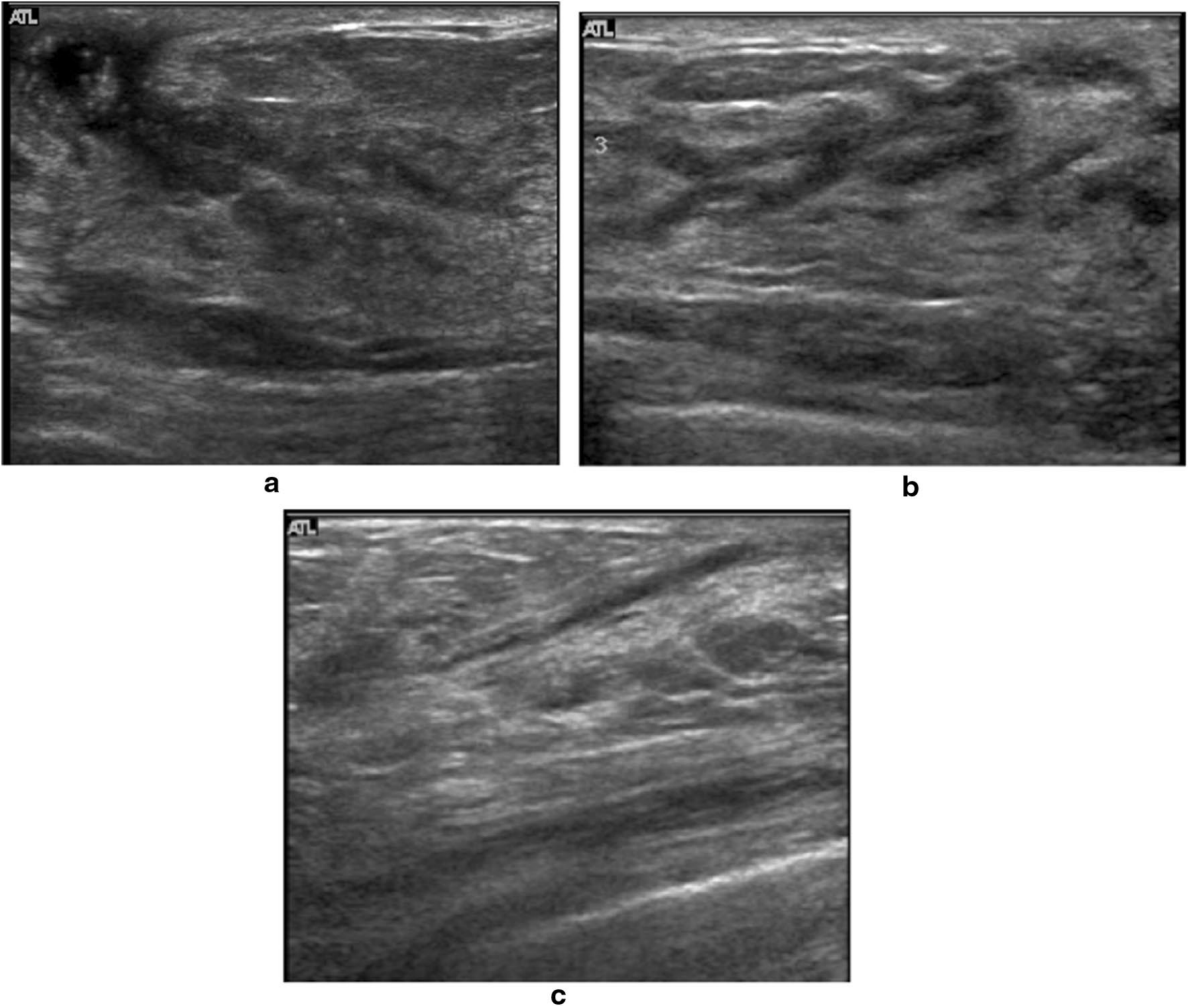
Three cases of ductal carcinoma in situ (DCIS). Ultrasound shows that the beginning is gentle or the solid component in the ducts is continuous

Note: Attention is required when there are internal echoes in the case of focal or segmental dilated ducts. If the starting point of the solid component in the dilated ducts is steep, there is a high likelihood of it being a benign lesion such as an intraductal papilloma (Fig. 5), and when the beginning is gentle or the solid component in the ducts is continuous, there is a high likelihood of it being a malignant lesion, such as DCIS (Fig. 6).

**Fig. 7:**
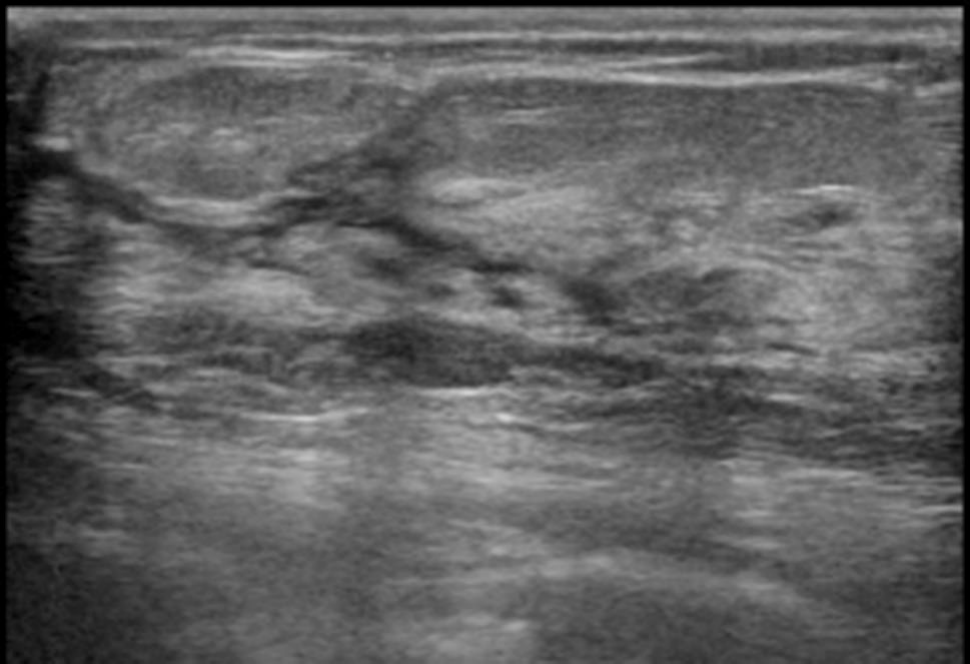
Case of ductal carcinoma in situ (DCIS). Ultrasound shows the irregularity of ductal caliber, but it is somewhat difficult to evaluate irregularity of ductal caliber based on static images alone

Note: It is somewhat difficult to evaluate irregularity of ductal caliber based on static images alone, but it is considered to be a finding that leans towards malignancy.

③ **Irregularity of ductal caliber (**Fig. 7**)**

### Architectural distortion

Definition: Findings of mammary gland structure-concentrated tightening/distortion at one spot or in a localized area in the mammary gland.

Architectural distortion, which is thought to be due to convergent changes in tissue, is found not only in malignant lesions but also in benign lesions.

**Fig. 8:**
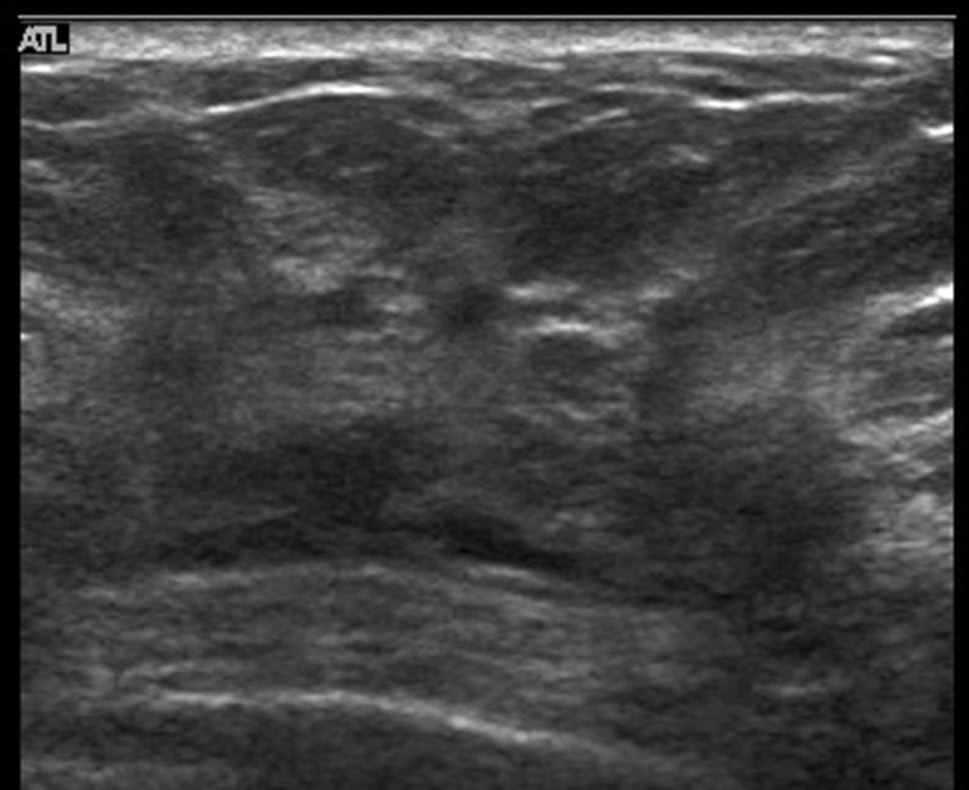
Case of invasive lobular carcinoma. Ultrasound shows the architectural distortion, but it is somewhat difficult to evaluate the architectural distortion

**Fig. 9:**
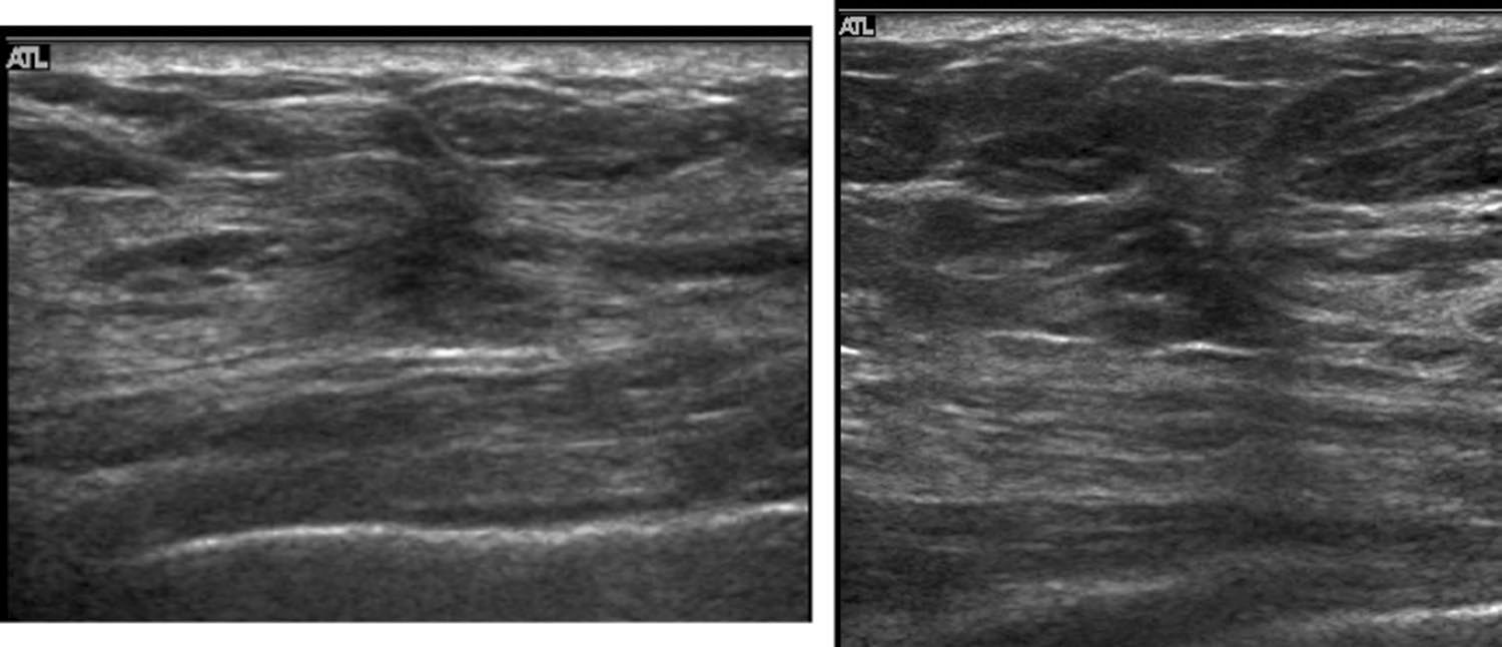
Both of these two cases with architectural distortion are ductal carcinoma in situ (DCIS)

Examples of lesions that present architectural distortion are IDC (scirrhous type), ILC (Fig. 8), and DCIS (Fig. 9) in the case of malignant lesions.

**Fig. 10:**
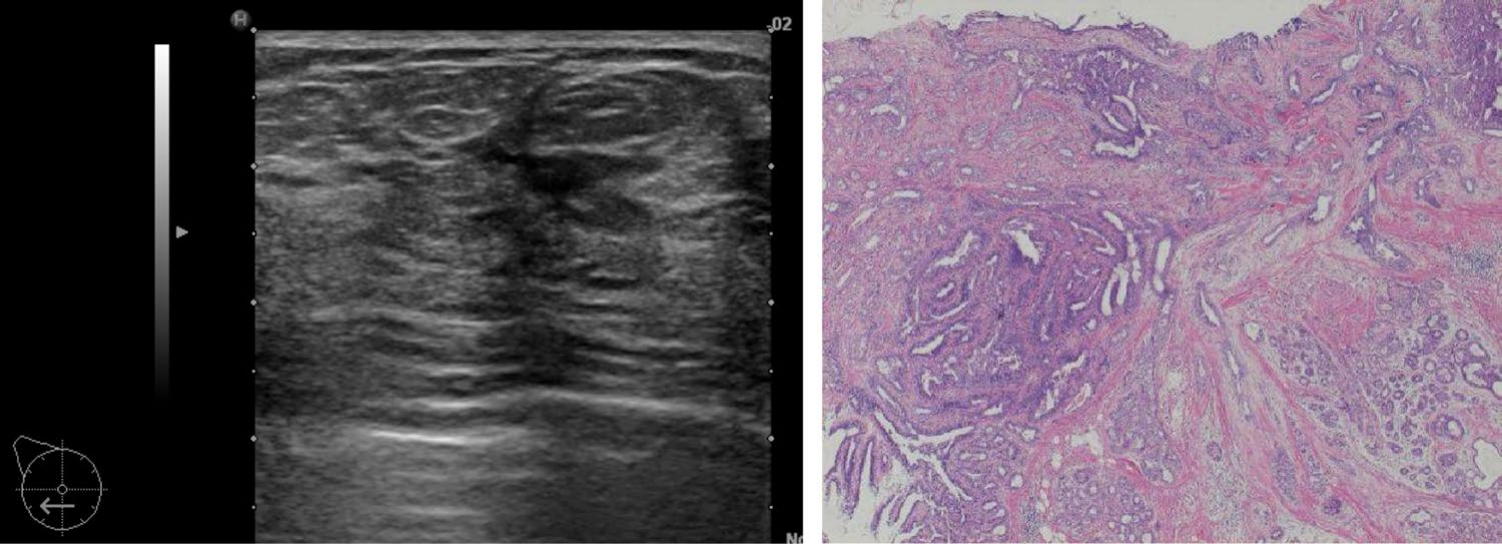
Case of radial sclerosing lesion. Ultrasound shows the architectural distortion

**Fig. 11:**
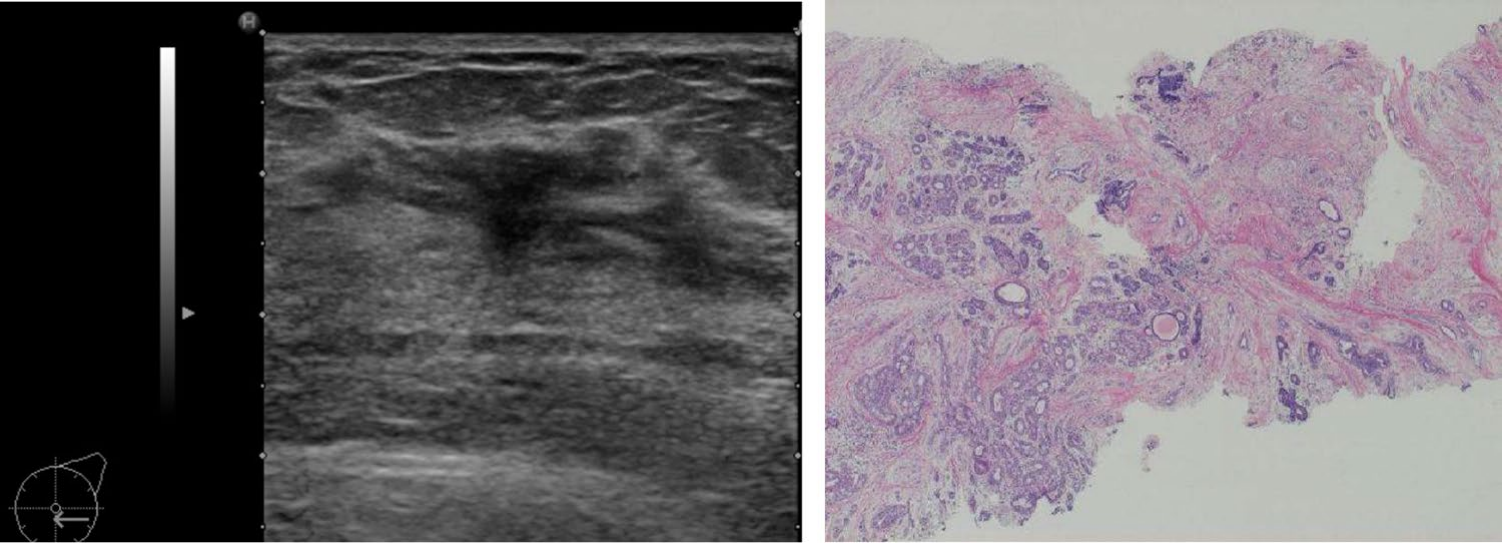
Case of radial sclerosing lesion. Ultrasound shows the architectural distortion

Other lesions that present architectural distortion are progression of breast cancer to Cooper's ligaments, and fibrosis after preoperative drug therapy in the case of malignant lesions, and radial scar (Fig. 10), sclerosing adenosis (Fig. 11), and postoperative scar in the case of benign lesions.

### Multiple small cysts

Definition: A finding in which multiple lesions recognized as small cysts several millimeters in size are observed in the mammary gland.

They are small hypoechoic masses that cannot be confirmed to be cysts (internal anechoic area), and they include masses that cannot be confirmed to be solid masses such as those without internal blood flow. In cases where clustering of microcysts is observed, the term "clustered microcysts" can be used.

A detailed examination is not needed when screening reveals multiple small cysts alone. This is because the frequency of malignancy is extremely low when the only finding is multiple small cysts (clustered microcysts), because they are often indolent even if malignant.

**Fig. 12:**
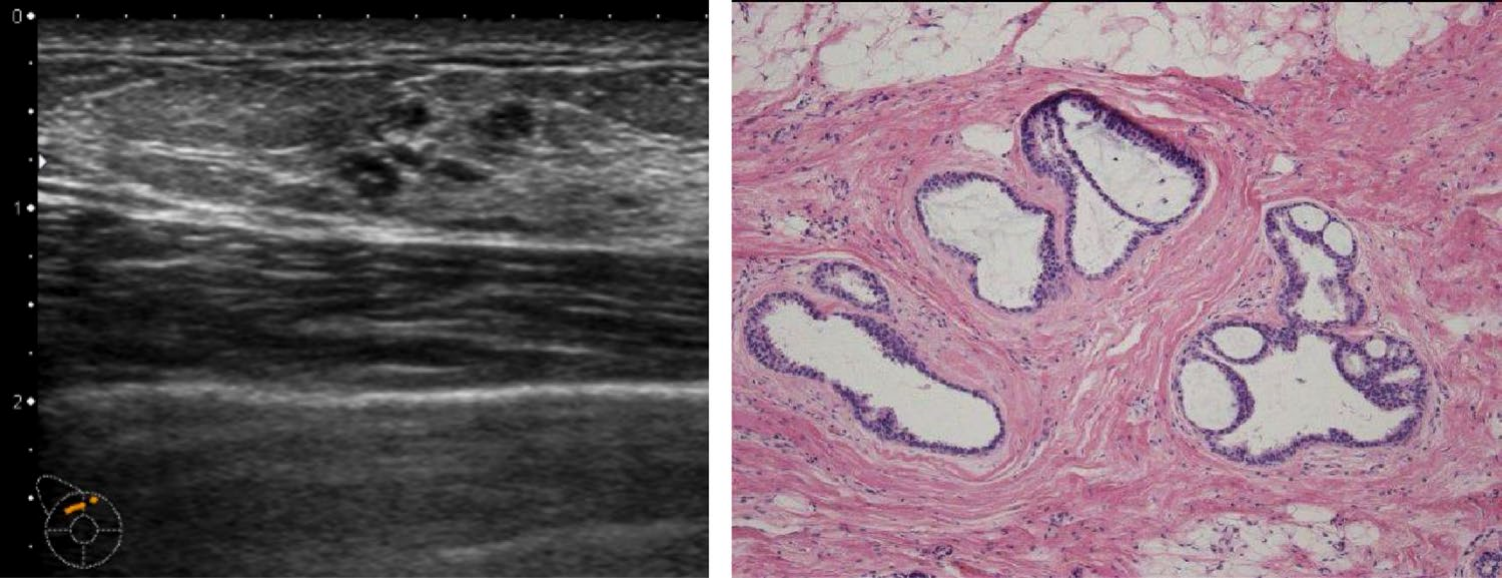
Ultrasound shows the multiple small cysts with a hypoechoic area. Histopathologic findings show ductal carcinoma in situ (DCIS), flat type

**Fig. 13:**
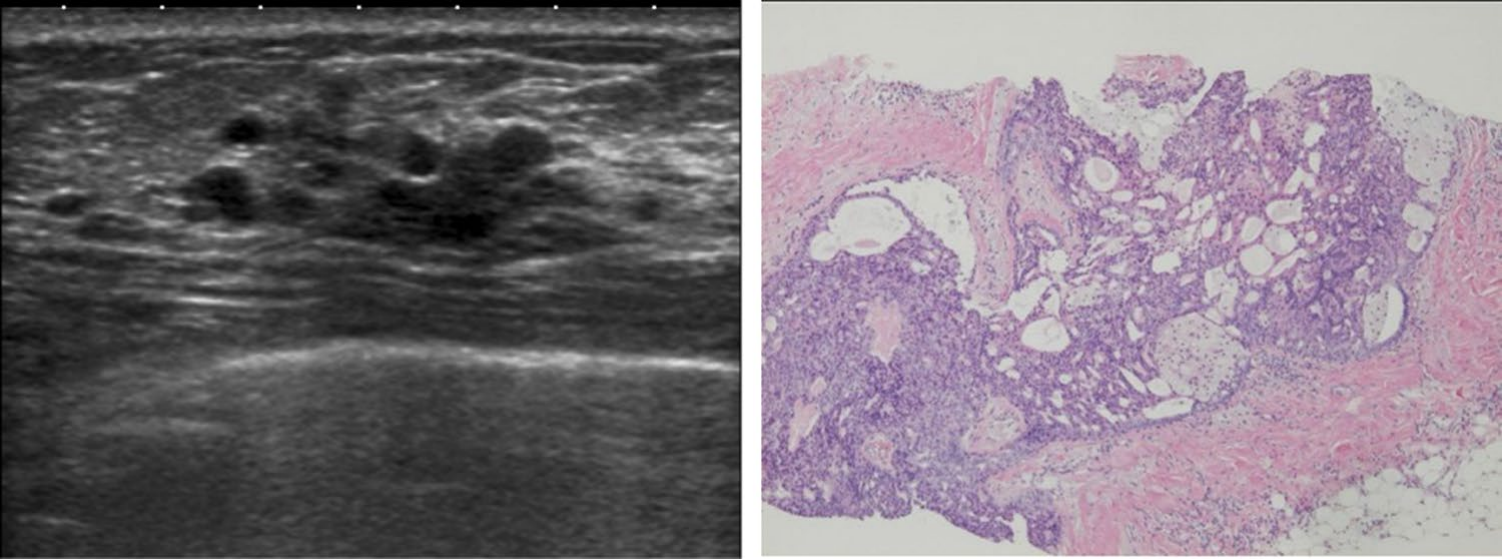
Ultrasound shows a hypoechoic area around multiple small cysts, but histopathological findings show duct papillomatosis

In rare cases, they indicate the presence of DCIS (Fig. 12), but most cases are benign lesions, such as apocrine metaplasia (Fig. 13).

### Echogenic foci without a hypoechoic area (Fig. 14)

**Fig. 14:**
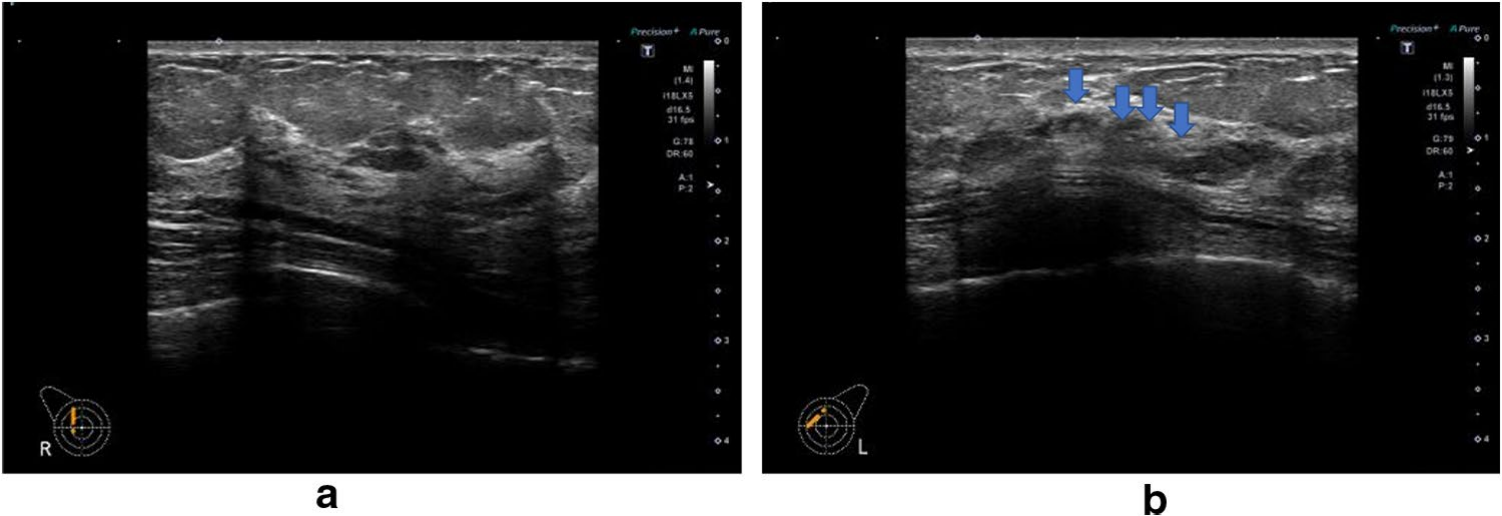
**a** Ultrasound shows the normal portion of the contralateral right breast. **b** Echogenic foci (arrow) corresponding to calcifications on mammography are seen in area A of the left breast on ultrasound

Definition: A lesion in which multiple hyperechoic foci thought to be microcalcifications are present focally or segmentally in the mammary gland, without involvement of a clear hypoechoic area or abnormalities of the ducts in the surrounding area.

This finding is not an independent US finding. As a rule, it is predicated on observation of calcifications that requires differentiation of benignancy/malignancy using mammography. It is used in cases, where the site of hyperechoic foci on US does not contradict the site of calcifications on mammography.

The presence of this finding may indicate DCIS, but they are helpful in determining the target for needle biopsy and other procedures.

6) The following are ancillary findings.

- Echogenic foci: Echogenic foci are defined as multiple point-like hyperechogenicity within the hypoechoic area or within the solid part of the abnormalities of the ducts. The presence of echogenic foci, suggesting multiple calcifications within the lesion, is an important finding, because it increases the likelihood of malignancy.
- Vascularity: When color Doppler clearly detects a large number of blood flow signals in or near the lesion, it is a helpful finding to suspect malignancy.
  Comparison with the contralateral mammary gland serves as a reference in the assessment.
- Elasticity: Malignant lesions often have increased stiffness over the surrounding tissue, which is helpful in distinguishing between benign from malignant.

### Lesion distribution

As for lesion distribution, assess and record the lesion site in the following order.

① Bilateral, unilateral
② Focal (clustered), segmental, diffuse

It is easy to grasp segmental distribution when making the assessment by envisioning an image that extends radially with the nipple as the peak. The term clustered is sometimes used for multiple small cysts.

A bilateral or diffuse (scattered) distribution usually indicates a benign lesion. Attention is required in the case of a segmental or focal (clustered) distribution as it may indicate malignant potential. In the case of a distinct segmental distribution, in particular, the possibility of DCIS should be considered. However, breast cancer that presents a finding of multiple small cysts alone is very rare even if the distribution is segmental or focal (clustered), and it is not thought to affect the vital prognosis; therefore, thorough examination is not necessary when screening does not reveal other findings such as hypoechoic area in the mammary gland and abnormalities of the ducts [9–11].

## Conclusion

Lesions that present non-mass abnormality findings on US include a variety of lesions, from benign to highly malignant breast cancers. The JSUM Terminology/Diagnostic Criteria Committee has classified US findings of non-mass abnormalities into five subtypes. Diagnostic accuracy may be improved by keeping in mind the classification and distribution of non-mass abnormalities when diagnosing lesions presenting as non-mass abnormalities.

## Data Availability

Data sharing is not applicable to this article as no datasets were generated or analyzed during the current study.
